# A flavin-dependent monooxgenase confers resistance to chlorantraniliprole in the diamondback moth, *Plutella xylostella*

**DOI:** 10.1016/j.ibmb.2019.103247

**Published:** 2019-12

**Authors:** Mark Mallott, Sarah Hamm, Bartlomiej J. Troczka, Emma Randall, Adam Pym, Charles Grant, Simon Baxter, Heiko Vogel, Anthony M. Shelton, Linda M. Field, Martin S. Williamson, Mark Paine, Christoph T. Zimmer, Russell Slater, Jan Elias, Chris Bass

**Affiliations:** a College of Life and Environmental Sciences, Biosciences, University of Exeter, Penryn Campus, Penryn, Cornwall, UK; b School of BioSciences, The University of Melbourne, Melbourne, VIC, 3010, Australia; c Department of Entomology, Max Planck Institute for Chemical Ecology, Jena, Germany; d Department of Entomology, Cornell University AgriTech, Geneva, NY, USA; e Department of Biointeractions and Crop Protection, Rothamsted Research, Harpenden, UK; f Liverpool School of Tropical Medicine, Liverpool, UK; g Syngenta Crop Protection, Werk Stein, Schaffhauserstrasse, Stein, Switzerland

**Keywords:** *Plutella xylostella*, Flavin monooxygenase, Resistance, Chlorantraniliprole, Diamide, Insecticides

## Abstract

The diamondback moth, *Plutella xylostella*, is a damaging pest of cruciferous crops, and has evolved resistance to many of the insecticides used for control, including members of the diamide class. Previous work on the molecular basis of resistance to diamides has documented mutations in the target-site, the ryanodine receptor, in resistant populations of *P. xylostella* worldwide. In contrast the role of metabolic resistance to this insecticide class is significantly less clear. Here we show that overexpression of a flavin-dependent monooxgenase (FMO) confers resistance to the diamide chlorantraniliprole in *P. xylostella*. Transcriptome profiling of diamide resistant strains, with and without target-site resistance, revealed constitutive over-expression of several transcripts encoding detoxification enzymes compared to susceptible strains. Two of these, *CYP6BG1*, and *PxFMO2* were particularly highly overexpressed (33,000 and 14,700-fold, respectively) in a resistant strain (HAW) lacking target-site resistance. After 17 generations without diamide selection the resistance of the HAW strain fell by 52-fold and the expression of *PxFMO2* by > 1300-fold, however, the expression of *CYP6BG1* declined by only 3-fold. Generation of transgenic *Drosophila melanogaster* expressing these genes demonstrated that *PxFMO2*, but not *CYP6BG1*, confers resistance *in vivo*. Overexpression of *PxFMO2* in the HAW strain is associated with mutations, including a putative transposable element insertion, in the promoter of this gene. These enhance the expression of a reporter gene when expressed in a lepidopteran cell line suggesting they are, at least in part, responsible for the overexpression of *PxFMO2* in the resistant strain. Our results provide new evidence that insect FMOs can be recruited to provide resistance to synthetic insecticides.

## Highlights

• Resistance to chlorantraniliprole in *Plutella xylostella* is associated with the overexpression of detoxification genes.
• Two transcripts encoding CYP6BG1 and PxFMO2 are overexpressed >14,000-fold in a resistant strain from Hawaii (HAW).
• PxFMO2 but not CYP6BG1 confer resistance to chlorantranilprole when expressed in transgenic *D. melanogaster.*
• In the absence of selection the resistance of the HAW strain fell by 52-fold and the expression of *PxFMO2* by > 1000-fold.
• Mutations (including a repetitive element insertion) in the *PxFMO2* promoter of the HAW strain enhance expression.

## Graphical abstract

**Figure:**
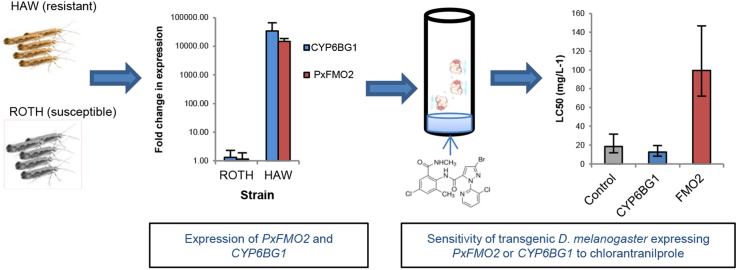

## 1 Introduction

The diamondback moth, *Plutella xylostella*, is one of the most destructive insect pests of cruciferous crops worldwide resulting in estimated costs to the global economy of US$4–5 billion per annum (Zalucki et al., 2012). In attempts to minimise crop damage caused by this pest growers have relied heavily on the use of synthetic insecticides for control. However, their intensive use, particularly in Asia, has resulted in the emergence of potent resistance to a range of different insecticide classes (Furlong et al., 2013). The diamide insecticides, comprising flubendiamide, chlorantraniliprole and cyantraniliprole, are a relatively new class of insecticide that were introduced to control *P. xylostella* from 2007 (Nauen, 2006; Troczka et al., 2017). They initially proved extremely effective at controlling this pest, in part, because they were not compromised by resistance mechanisms that had evolved to older compounds. However, just 18 months after the introduction of flubendiamide, populations of *P. xylostella* with tolerance to this compound were reported in Thailand (Sukonthabhirom et al., 2011). In the years following, diamide resistance was reported in populations from the Philippines, Taiwan, India, China, Brazil and the US (Troczka et al., 2017).

Investigation of the mechanistic basis of resistance to diamides has primarily focussed on insensitivity of the target-site of this insecticide class: the ryanodine receptor (RyR), a ligand-gated calcium channel located in the sarco- and endoplasmic reticulum of neuromuscular tissues. Sequencing of the putative ligand binding regions of the RyR from strains of *P. xylostella* from Thailand and the Philippines identified a mutation associated with diamide resistance that results in a G4946E amino acid substitution (Troczka et al., 2012). Subsequent work on a resistant *P. xylostella* population from China identified additional substitutions, E1338D, Q4594L and I4790M, associated with resistance (Guo et al., 2014). Since these reports, radioligand binding studies and CRISPR-Cas genome editing have provided unequivocal functional evidence that two of the substitutions, G4946E and I4790M, alter the affinity of the RyR for diamides and confer resistance (Douris et al., 2017; Steinbach et al., 2015; Troczka et al., 2015).

In contrast to the comprehensive characterisation of target-site resistance to diamides, the role and underpinning mechanisms of metabolic resistance to this insecticide class is less well understood. Studies of metabolic resistance to insecticides more generally have most frequently implicated the overexpression of insect enzymes belonging to three main superfamilies, namely cytochrome P450s (P450s), glutathione-s-transferases (GSTs), and carboxyl/cholinesterases (CCEs) (Li et al., 2007). In *P. xylostella* several studies have used inhibitors, enzyme assays, or transcriptome profiling to implicate these enzyme families in resistance to diamides (Hu et al., 2014b; Kang et al., 2017; Lin et al., 2013; Liu et al., 2015; Wang et al., 2013). However, none of these established a clear functional association between these enzymes and resistance. In contrast, two studies investigating chlorantraniliprole resistance in *P. xylostella* populations from China implicated the overexpression of the P450s *CYP6BG1* and *CYP321E1* in resistance (Hu et al., 2014a; Li et al., 2018). RNA interference provided supporting evidence that both P450s contribute to chlorantraniliprole resistance (Hu et al., 2014a; Li et al., 2018), with transgenic overexpression of *CYP6BG1* providing additional evidence of its causal role in resistance (Li et al., 2018). Beyond GSTs, P450s and CCEs, only a single study of metabolic resistance has associated alternative families of detoxification enzymes in the resistance of *P. xylostella* to diamides, with the UDP-glycosyltransferase gene *UGT2B17* overexpressed in chlorantraniliprole resistant *P. xylostella* strains from China (Li et al., 2017). RNAi knockdown of this gene increased sensitivity to this compound suggesting it contributes to resistance. Beyond *P. xylostella* recent work on the model insect *Drosophila melanogaster* using a systems genetics approach linked allelic variation in the neuromuscular gene *Stretchin Myosin light chain kinase* and enhanced expression of the P450 *Cyp12d1* with reduced sensitivity to chlorantraniliprole (Green et al., 2019).

In the current study we used transcriptome profiling, in combination with functional analysis of candidate genes, to explore the role of metabolic resistance to diamides in strains of *P. xylostella* from Thailand and Hawaii.

## 2 Materials and methods

### 2.1 Insect strains

The ROTH insecticide susceptible strain of *P. xylostella* (originally from the Philippines) has been maintained at Rothamsted Research under laboratory conditions for more than 30 years without insecticide selection. The HAW strain was collected in Hawaii and initially reared at Cornell University. At generation G3 it was selected with 0.5 ppm chlorantraniliprole with the selecting dose increased every few generations reaching 10 ppm at G30, at this point the strain was transferred to Rothamsted Research where it was selected approximately every other generation with a maintenance dose of 6–8 ppm chlorantraniliprole. Samples of this strain were taken for molecular analysis at G41 and initial insecticide bioassays (data shown in Table 1) performed on G45, the stability experiment (section 2.6) was performed from G71 to G87. The CM strain is a field-collected susceptible strain from Chiang Mai Thailand, and the CHL and FLU strains are field-collected diamide resistant strains collected from Bang Bua Thong Thailand. All three strains were reared at Syngenta. The CM strain corresponds to the ThaiS strain and the CHL strain to the ThaiR strain described previously (Troczka et al., 2012). The CHL and FLU strains were selected approximately every two weeks with a maintenance dose of 10 ppm chlorantraniliprole and flubendiamide respectively. All strains were reared at 23 ± 1 °C, 70% relative humidity. Larvae were fed on cabbage plants (*Brassica oleracea*) and adults on a 10% (w/v) sucrose solution.

**Table 1:** Log-dose probit-mortality data for two diamide insecticides tested against four strains of *P. xylostella* by leaf-dip bioassay. Data for the CHL and CM strain is taken from (Troczka et al., 2012). Resistance ratio (RR) of the HAW strain is relative to the lab susceptible ROTH strain. The RR of the CHL and FLU strains is relative to the CM strain. For information the frequency of the G4946E mutation of each strain is shown in column 2.

Strain	G4946E frequency	Chlorantraniliprole			Flubendiamide		
		LC_50_-value [mg/L^−1^]	95% CL	RR	LC_50_-value [mg/L^−1^]	95% CL	RR
HAW	0	12.5	4.70–34.00	104	2.45	1.31–4.56	9
ROTH	0	0.12	0.03–0.50	–	0.26	0.14–0.50	–
CHL	1	>60	–	>200	>60	–	>750
FLU	1	797	730–881	2657	>200	–	>2500
CM	0.05	0.3	0.25–0.38	–	0.08	0.06–0.11	–

### 2.2 Insecticide bioassays

Technical grade insecticides (purity >98%) were obtained from Sigma Aldrich. Stock solutions were prepared in acetone and diluted using an aqueous solution of 0.1% (w/v) Triton-X100. At least six concentrations with three replicates were tested for each insecticide. Individual cabbage leaf disks 65 mm in diameter were dipped in an insecticide solution for 10 s, dried on paper towel and then transferred to petri dishes containing a water-moistened filter paper. Ten second instar larvae were placed on each leaf and petri dishes stored at 22 ± 1 °C, 60% relative humidity and ambient photoperiod. Larvae were scored for mortality after 72 h and were assessed as dead if no movement was observed following repeated agitation with a fine paintbrush. Control larvae feeding on aqueous Triton X-100-treated leaves showed <10% mortality in all bioassays. Raw bioassay data was corrected for any control mortality using Abbott's formula (Abbott, 1925) then analysed by probit regression to determine LC_50_ values (the concentration expected to kill 50% of the tested individuals) and confidence limits using GenStat (18th Edition, VSN International). Where 95% confidence intervals did not overlap, populations were considered to be significantly different in sensitivity to the test compound.

### 2.3 Genotyping *P. xylostella* for target-site alterations in the ryanodine receptor

Total RNA was extracted from individual 3rd/4th instar larvae or pools of 10 larvae using the ISOLATE RNA mini kit (Bioline Reagents Ltd., UK) following the manufacturer's protocol. cDNA was synthesised from 4 μg of total RNA using Superscript III reverse transcriptase (Invitrogen, USA) and random hexamers according to the manufacturer's protocol. Amplification of the regions of the ryanodine receptor encompassing the sites of the previously described mutations was carried out using the primers and protocol described previously (Troczka et al., 2012). PCR products were purified using the Wizard SV Gel and PCR Clean-up System (Promega, USA) according to the manufacturer's protocol and sequenced directly by Eurofins Genomics. Sequencing data were analysed using Geneious v7 Software (Biomatters Ltd., NZL).

### 2.4 Microarray analysis

The microarray used in this study was designed using the Agilent eArray platform (Agilent Technologies, USA) by the Heiko Vogel lab and was developed from 46,280 expressed sequence tag (EST) contigs. Two probes were designed for all contigs and then the array filled with replica probes. The final slide layout consisted of four arrays of 180,000 60-mer probes produced by *in situ* oligonucleotide synthesis. Total RNA was extracted from four pools of 5 fourth instars as described above and 200 ng used to generate labelled cRNA, which was hybridized to arrays and washed as described in Agilent's Quick Amp Labelling Protocol (Version 6.5). Each microarray experiment consisted of four biological replicates and incorporated a dye swap design whereby the Cy3 and Cy5 labels were swapped between resistant and susceptible strains. Microarrays were scanned with an Agilent G2505C US10020348 scanner, and fluorescent intensities of individual spots were obtained using the Agilent Feature Extraction software with default Agilent parameters. Data normalization, filtering, dye flipping and statistical analysis were performed using the GeneSpring GX 11 suite (Agilent). For statistical analysis, a *t*-test against zero using the Benjamini-Hochberg false discovery rate (FDR) method for multiple testing correction was used to detect significantly differentially expressed genes. Genes meeting a corrected p value cut-off of 0.05 and showing a transcription ratio >2-fold in either direction were considered to be differentially transcribed between resistant and susceptible strains. Lists of differentially expressed genes were compared using jvenn (Bardou et al., 2014).

### 2.5 Quantitative PCR

Quantitative RT-PCR was used to validate microarray data by examining the expression profile of candidate resistance genes. Primers were designed to amplify a fragment of 90–150 bp in size and are listed in Table S1. Total RNA and cDNA were prepared as described above with PCR reactions (20 μL) containing 4 μL of cDNA (10 ng), 10 μL of SensiMix SYBR Kit (Bioline), and 0.25 μM of each primer. Samples were run on a Rotor-Gene 6000 (Corbett Research, France) using temperature cycling conditions of: 10 min at 95 °C followed by 40 cycles of 95 °C for 15 s, 57 °C for 15 s and 72 °C for 20 s. A final melt-curve step was included post-PCR (ramping from 72°C to 95 °C by 1 °C every 5 s) to confirm the absence of any non-specific amplification. The efficiency of PCR for each primer pair was assessed using a serial dilution of 100 ng–0.01 ng of cDNA. Each qRT-PCR experiment consisted of three independent biological replicates with two technical replicates for each. Data were analysed according to the ΔΔCT method (Pfaffl, 2001), using the geometric mean of two selected housekeeping genes (actin and glyceraldehyde-3-phosphate dehydrogenase) for normalization according to the strategy described previously (Vandesompele et al., 2002). Significant differences in gene expression in qPCR experiments were determined using unpaired t-tests for pair-wise comparisons and one-way ANOVA with post hoc testing (Tukey HSD) for comparisons of more than two samples.

### 2.6 Stability of resistance in the absence of selection

Starting from a selected population (8 ppm chlorantraniliprole) of the HAW strain, 2nd generation post-selection (G0) offspring were separated into two lines. The ‘selected’ line was subject to 6 ppm chlorantraniliprole selection every 2nd or 3rd generation, while the ‘unselected’ line was untreated. Approximately every other generation, insecticide bioassays were performed (as described above) and a subsample of 40 individuals (4 cohorts of 10 individuals) snap frozen in liquid nitrogen for molecular analyses. Upon conclusion of the experiment, RNA was extracted and cDNA synthesised from each of the frozen cohorts, and the expression of candidate resistance genes examined by qPCR as described above.

### 2.7 Transgenic expression of candidate genes in *D. melanogaster*

Prior to expression the full coding sequence of candidate genes was verified by PCR and sequencing using the primers detailed in Table S1 following the methods detailed in section 2.3. Candidate resistance genes were then synthesised (GeneArt, ThermoScientific, USA) and cloned into the pUASTattB plasmid (GenBank: EF362409.1). Using the PhiC31 system, constructs were transformed into the germline of a *D. melanogaster* strain carrying an attP docking site on chromosome 2 (attP40) and the phiC31 integrase gene under the control of the vasa regulatory region on the X chromosome [y w M(eGFP, vas-int, dmRFP)ZH-2A; P{CaryP}attP40] (Markstein et al., 2008). The transgenic lines obtained were balanced and the integration of genes confirmed by PCR and sequencing using Phusion DNA polymerase (Thermo, USA) as described previously (Manjon et al., 2018) with the primers detailed in Table S1. Virgin females of the Act5C-GAL4 strain were crossed with UAS-gene-of-interest males. Bioassays were used to assess the susceptibility of adult female flies to chlorantraniliprole or flubendiamide. Several concentrations were overlaid onto 1.5% agar containing 1% sucrose in standard Drosophila vials and allowed to dry overnight at room temperature. 10-15 adult flies (two to five days post eclosion) were then added to each vial and mortality assessed after 72 h. Four replicates were carried out for each concentration. Control mortality was assessed using vials containing agar/sucrose minus insecticide. LC_50_ values and 95% fiducial limits were calculated as above.

### 2.8 Promoter sequencing and reporter gene assays

Promoter fragments were amplified by PCR using Phusion high fidelity DNA polymerase (New England BioLabs, USA) in a nested PCR approach using primers Px_FMOPromSacI_F1 and Px_FMOProm_R1 in a first round of PCR and Px_FMOProm_F2 and Px_R_FMOPromNcoI_R2 for amplification from the ROTH strain, and primers Px_FMOProm_F2 and Px_HS_FMOPromNcoI_R2 for amplification from the HAW strain (see Table S1). The SacI and NcoI restriction sites incorporated by these primers were used to ligate fragments into the pGL3-basic luciferase reporter vector (Promega), with constructs then transformed into DH5α competent cells (Invitrogen). Plasmids were extracted with the GeneJet plasmid miniprep kit (ThermoScientific, USA), sequenced and then adjusted to 400 ng/μl for use in dual luciferase assays using the Sf9 insect cell line. Approximately 1 × 10^6^ cells per well were plated into 6-well plates 2 h prior to transfection and allowed to reach 60%–70% confluency. Insect GeneJuice transfection reagent (Novagen, Germany) was used for transfection of constructs and the Dual-Luciferase Reporter Assay (Promega) used for promoter activity measurements according to the manufacturer's protocols. 2 μg of reporter constructs and pGL3 without insert (as a control) were co-transfected with 4 ng Renilla luciferase pRL-CMV using GeneJuice and incubated at 27 °C. 4 hr post-transfection, the transfection mixture was removed and replaced with supplemented Grace's Insect Medium (ThermoScientific). Following further incubation at 27 °C for 48 h and washing of cells with PBS, cells from each well were harvested in 500 μL passive lysis buffer (Promega) and luciferase activity measured on a GloMax 20/20 (Promega). Construct luciferase activity was normalised to Renilla luciferase activity as described in the manufacturer's protocol.

### 2.9 Data availability

The sequences characterised in this study have been deposited in NCBI under accessions KY924612-KY924617.

## 3 Results

### 3.1 Sensitivity of *P. xylostella* strains to diamide insecticides

The HAW strain exhibited significant resistance to chlorantraniliprole (resistance ratio (RR) of >100) but only modest tolerance to flubendiamide (RR < 10) compared to the laboratory reference strain ROTH in leaf-dip insecticide bioassays (Table 1). In comparison CHL and FLU, both field collected resistant strains from Thailand, had LC_50_ values greater than 60 mg/L for both chlorantraniliprole and flubendiamide, resulting in resistance ratios of >200 compared to the CM field-collected susceptible strain from Thailand.

### 3.2 The resistant HAW strain lacks target-site alterations previously associated with diamide resistance

As detailed in the introduction, resistance to diamides has been previously linked to mutations (G4946E and I4790M) in the membrane-spanning domain of the RyR in *P. xylostella* (Guo et al., 2014; Troczka et al., 2012), and the frequency of these mutations in the CHL and CM strains have been determined previously (Troczka et al., 2012). The CHL strain was found to be fixed for the G4946E substitution, explaining, at least in part, the high resistance of this strain to diamide insecticides. In contrast, this mutation was observed at a frequency of just 0.05 and in the heterozygous state in the CM strain. To examine if G4946E or I4790M are present in the HAW or FLU strains sequences of the RyR encompassing these mutation sites were obtained from cDNA derived from pools of several larvae and 12 individual larvae. In the FLU strain, all the sequences were homozygous for the E4946 allele, indicating this strain is fixed for the G4946E substitution; in contrast, no sequences were observed with the I4790M substitution. In the case of the HAW strain, all the sequences obtained encoded the wild-type (susceptible) amino acids (glycine and isoleucine) at positions 4946 and 4790 respectively. Thus, the resistance of the HAW strain to diamides is not explained by known target-site alterations in the RyR.

### 3.3 Transcriptome profiling identifies candidate detoxification genes in diamide resistant *P. xylostella*

**Fig. 1:**
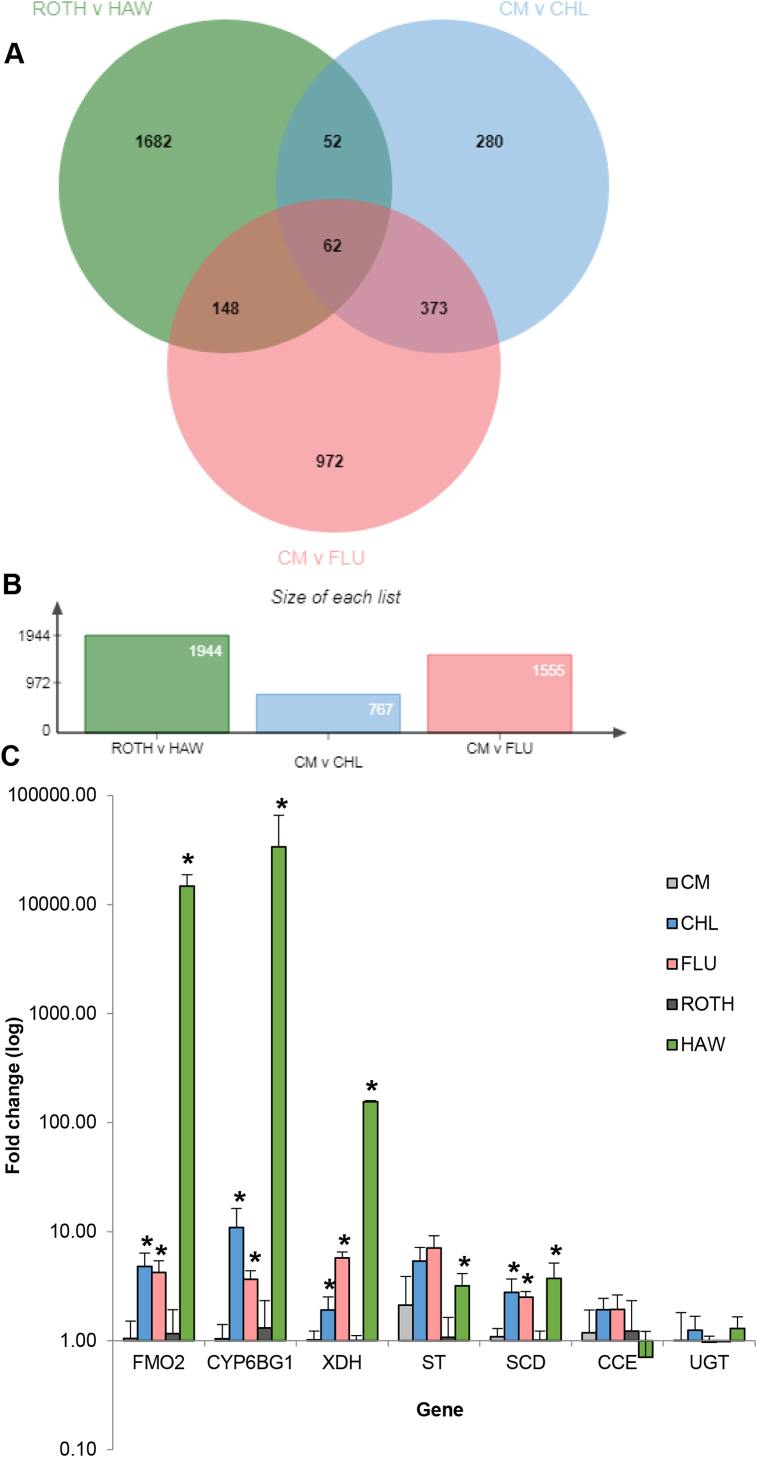
**Transcriptome profiling of diamide resistant and susceptible *P. xylostella* strains.** (A) Venn diagram showing numbers of differentially expressed genes in multiple microarray comparisons of *P. xylostella*. (B) Numbers of differentially expressed genes in each treatment comparison. (C) Relative expression (fold change) of candidate resistance genes in different strains of *P. xylostella* measured by qPCR. Error bars display 95% CLs. Significant differences (p < 0.05) in expression between resistant strains and the relevant susceptible reference strain is denoted using asterisks above bars as determined by One-way ANOVA with post hoc testing (Tukey HSD) (CM versus CHL and FLU) or unpaired t tests (ROTH versus HAW). ROTH: long-term lab susceptible strain, HAW: chlorantraniliprole resistant strain from Hawaii, CM: susceptible field strain from Thailand, CHL: diamide resistant field strain from Thailand, FLU: diamide resistant field strain from Thailand. XDH: xanthine dehydrogenase, ST: sugar transporter, SCD: short-chain dehydrogenase, CE: carboxylesterase.

Transcriptome profiling, using gene expression microarrays was used to identify candidate genes potentially involved in the enhanced metabolism of diamides in the resistant *P. xylostella* strains. Comparison of the HAW strain with the susceptible ROTH strain identified 5119 probes representing 4269 transcripts (of which 1944 had functional annotation) that were differentially expressed (DE) > two-fold (Table S2). Comparison of the CHL strain with the CM strain identified 1597 probes representing 1166 transcripts of which 767 had functional annotation (Table S3), and comparison of the FLU strain with the CM strain identified 3331 probes representing 2641 transcripts of which 1555 had functional annotation (Table S4). A total of 62 genes were DE in all three comparisons (Fig. 1A and B) of which just 29 were DE in the same direction in all comparisons (i.e. consistently upregulated or downregulated in all 3 resistant strains compared to the respective susceptible stains). The full list of these genes, along with fold-change values and a description based on the closest BLAST hit, is given in Table 2. Several transcripts encoding detoxification enzymes that metabolise xenobiotics were highly overexpressed in all three resistant strains compared to the respective susceptible strains. Among the most highly upregulated were two transcripts, Contig_25748 and Contig_42842, encoding a flavin-containing monoxgenase (FMO) overexpressed 69.8- and 28.9- fold in the HAW strain, 7.6- and 17.1-fold in the CHL strain and 4.5- and 8.6-fold in the FLU strain. Both transcripts were most similar to a single FMO of *Bombyx mori*, FMO2 (65.4% and 60.8% identity at the nucleotide level), and shared 46 bp of overlapping sequence, with subsequent PCR and sequencing confirming they are encoded by the same gene. Only a single transcript (Contig_42837) was overexpressed in all array comparisons that has been previously implicated in diamide resistance in *P. xylostella* (Li et al., 2018). This transcript encodes the P450 CYP6BG1 and was overexpressed 13.2-, 11-, and 3.3-fold in the HAW, CHL and FLU strains compared to the respective susceptible strains. Other transcripts encoding putative detoxification enzymes included Contig_33985, a caboxylesterase (overexpressed 43.7-, 6.1-, and 5.9-fold in the HAW, CHL and FLU strains), Contig_42163, a short chain dehydrogenase (SCD) (overexpressed 3.6-, 8.5-, 3.9-fold in the HAW, CHL and FLU strains), Contig_2422, encoding a uridine diphosphate-glucuronosyltransferases (UGT) (overexpressed 18.2-, 3.0- and 2.0-fold in the HAW, CHL and FLU strains) and Contig_1528, a xanthine dehydrogenase (XDH) (overexpressed 5.5-, 20.3-, and 2.7-fold in the HAW, CHL and FLU strains). Beyond detoxification genes, other potential candidates with a potential link to insecticide resistance were two transcripts (Contig_2416 and Contig_39731) encoding ATP-binding cassette transporters (ABC transporters) overexpressed 3.4–12.1-fold in the resistant strains. Both transcripts return the same BLAST hit (sugar transporter 4 of *Bombyx mori*) and share 97% sequence identity strongly suggesting they are alternative transcripts of the same gene.

**Table 2:** Candidate resistance genes identified as significantly differentially expressed in three different microarray comparisons of diamide resistant and susceptible *P. xylostella* strains. In the first comparison, the ROTH strain was compared to the HAW strain; in the second comparison the CM strain was compared to the CHL strain; in the third comparison the CM strain was compared to the FLU strain. The p values displayed are corrected for multiple hypothesis testing (Benjamini-Hochberg).

ContigID	ROTH vs HAW		CM vs CHL		CM vs FLU		BLAST hit description
	p-value	Fold change	p-value	Fold change	p-value	Fold change	
Contig_2909	0.0155	132.32	0.0146	2.26	0.0239	2.95	gi|91080871|ref|XP_972325.1|PREDICTED: similar to nidogen [*Tribolium castaneum*]
Contig_42182	0.0161	114.76	0.0067	8.82	0.0470	4.30	gi|170058287|ref|XP_001864856.1|2-oxoisovalerate dehydrogenase subunit beta, mitochondrial [*Culex quinquefasciatus*]
Contig_40035	0.0203	95.94	0.0413	4.41	0.0343	4.05	gi|170035152|ref|XP_001845435.1|luciferin 4-monooxygenase [*Culex quinquefasciatus*]
Contig_25748	0.0154	69.81	0.0414	7.62	0.0319	4.53	gi|297139712|ref|NP_001171912.1|flavin-dependent monooxygenase FMO2 [*Bombyx mori*]
Contig_3261	0.0243	56.39	0.0054	21.08	0.0396	11.69	gi|153791757|ref|NP_001093275.1|myo-inositol oxygenase [*Bombyx mori*]
Contig_19228	0.0154	45.96	0.0024	8.76	0.0410	5.37	gi|114052174|ref|NP_001040228.1|aminoacylase [*Bombyx mori*]
Contig_33985	0.0145	43.69	0.0009	6.14	0.0107	5.91	gi|257480055|gb|ACV60241.1|antennal esterase CXE14 [Spodoptera littoralis]
Contig_2963	0.0219	31.45	0.0003	12.17	0.0189	6.43	gi|114052174|ref|NP_001040228.1|aminoacylase [*Bombyx mori*]
Contig_2356	0.0141	30.82	0.0007	3.39	0.0139	2.12	gi|262530078|gb|ACY69180.1|phosphoglucomutase [Spodoptera exigua]
Contig_42842	0.0342	28.88	0.0099	17.19	0.0461	8.64	gi|297139712|ref|NP_001171912.1|flavin-dependent monooxygenase FMO2 [*Bombyx mori*]
Contig_1528	0.0223	19.67	0.0222	5.54	0.0498	2.70	gi|2282473|dbj|BAA21640.1|xanthine dehydrogenase [*Bombyx mori*]
Contig_2422	0.0316	18.18	0.0089	3.01	0.0084	2.00	gi|308316676|gb|ACZ97420.2|UGT35E1 [Zygaena filipendulae]
Contig_4134	0.0349	17.80	0.0352	3.02	0.0018	2.52	gi|170038788|ref|XP_001847230.1|phosphoglucomutase [*Culex quinquefasciatus*]
Contig_2518	0.0354	15.48	0.0184	3.40	0.0026	5.16	gi|170061127|ref|XP_001866102.1|conserved hypothetical protein [*Culex quinquefasciatus*]
Contig_42837	0.0333	13.19	0.0021	11.02	0.0128	3.30	gi|163256092|dbj|BAF95609.1|cytochrome P450 [*Plutella xylostella*] CYP6BG1
Contig_19431	0.0411	12.50	0.0081	2.80	0.0024	4.49	gi|156339309|ref|XP_001620137.1|hypothetical protein NEMVEDRAFT_v1g48053 [*Nematostella vectensis*]
Contig_2416	0.0195	9.66	0.0100	12.12	0.0307	3.44	gi|284813579|ref|NP_001165395.1|sugar transporter 4 [*Bombyx mori*]
Contig_4158	0.0231	9.37	0.0463	3.42	0.0052	2.38	gi|268306460|gb|ACY95351.1|ribosomal protein S15 [Manduca sexta]
Contig_39731	0.0179	7.07	0.0080	9.80	0.0170	3.70	gi|284813579|ref|NP_001165395.1|sugar transporter 4 [*Bombyx mori*]
Contig_3596	0.0370	6.25	0.0113	3.46	0.0137	2.46	gi|307204053|gb|EFN82952.1|Probable aspartate aminotransferase, cytoplasmic [Harpegnathos saltator]
Contig_42715	0.0149	5.84	0.0184	2.65	0.0431	2.32	gi|170041293|ref|XP_001848403.1|zinc finger protein [*Culex quinquefasciatus*]
Contig_1685	0.0326	4.78	0.0055	3.93	0.0024	2.40	gi|195108671|ref|XP_001998916.1|GI24228 [*Drosophila mojavensis*]
Contig_2566	0.0188	4.29	0.0009	5.43	0.0345	5.13	gi|208972529|gb|ACI32825.1|beta-1,3-glucan recognition protein 1 [Helicoverpa armigera]
Contig_42163	0.0173	3.65	0.0428	8.07	0.0440	3.93	gi|260908006|gb|ACX53802.1|hydroxybutyrate dehydrogenase [*Heliothis virescens*]
Contig_2648	0.0402	3.60	0.0017	3.25	0.0051	2.54	gi|91092064|ref|XP_970689.1|PREDICTED: similar to selenium-binding protein [*Tribolium castaneum*]
Contig_3271	0.0236	3.54	0.0070	3.28	0.0121	3.57	gi|112982980|ref|NP_001037090.1|serine protease inhibitor 4 [*Bombyx mori*]
GM003070.1	0.0376	3.19	0.0027	2.90	0.0411	2.31	ref|XM_001978934.3|PREDICTED: *Drosophila erecta* protein transport protein Sec23A (LOC6552784), transcript variant X5, mRNA
Contig_1694	0.0151	2.10	0.0490	10.85	0.0037	11.86	gi|157117489|ref|XP_001658792.1|3-hydroxyacyl-coa dehyrogenase [*Aedes aegypti*]
Contig_36865	0.0229	−4.28	0.0019	−2.30	0.0166	−2.78	gi|114052156|ref|NP_001040222.1|thioredoxin family Trp26 [*Bombyx mori*]

Real-time quantitative PCR was used to validate the microarray results by examining the expression profile of the candidate genes. Out of the seven genes tested QPCR validated the significant overexpression of four, *PxSCD*, *PxXDH*, *CYP6BG1* and *PxFMO2*, in both resistant strains compared to the respective susceptible strains (Fig. 1C). The latter two genes were particularly highly expressed in the HAW strain with *PxFMO*2 overexpressed 14,700-fold and *CYP6BG1* 33,000-fold compared to the ROTH strain. These expression ratios are much higher than those generated in microarray experiments, however, this finding is consistent with other studies which have also reported that the Agilent array platform frequently underestimates the true expression level of genes that are highly expressed (Marcombe et al., 2009; Puinean et al., 2010).

### 3.4 Expression of *PxFMO2* but not *CYP6BG1* confers resistance to chlorantraniliprole *in vivo*

**Fig. 2:**
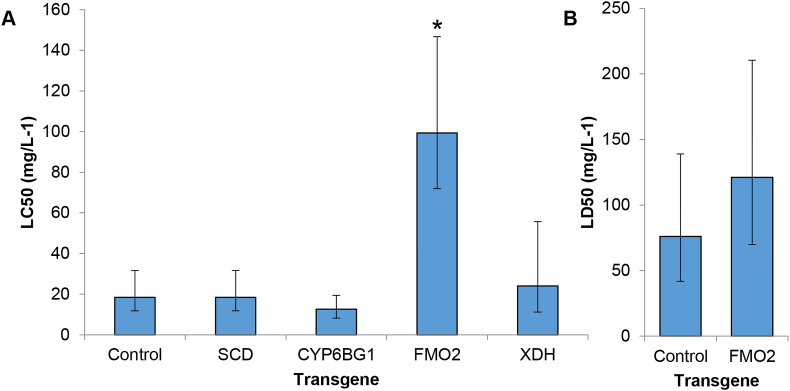
**Insecticide bioassays of transgenic *D. melanogaster* expressing *P. xylostella* candidate resistance genes to select diamide insecticides.** A) Sensitivity of transgenic flies to chlorantraniliprole. B) Sensitivity of transgenic flies to flubendiamide. XDH: xanthine dehydrogenase, SCD: short-chain dehydrogenase. Error bars indicate 95% CLs. Significant changes in sensitivity of control and transgenic lines are indicated using an asterisk and are based on non-overlapping 95% fiducial limits of LC_50_ values.

To functionally validate the role of the candidate genes identified by transcriptome profiling and qPCR in chlorantraniliprole resistance a panel of transgenic lines of *D. melanogaster* were created expressing *PxFMO2*, *CYP6BG1*, *PxSCD* or *PxXDH*. Compared to control flies (of the same genetic background but without a transgene), flies expressing *PxSCD*, *PxXDH* or *CYP6BG1* showed no resistance to chlorantraniliprole in insecticide bioassays (Fig. 2A). In contrast, flies expressing *PxFMO2* exhibited significant (7.9-fold) resistance to this compound compared with control flies demonstrating unequivocally that expression of *PxFMO2* is sufficient to confer a resistant phenotype to this compound (Fig. 2A). Because the *P. xylostella* HAW strain, which expresses *PxFMO2* at very high levels (Fig. 1C), shows only low levels of resistance to flubendiamide (Table 1), we also examined the sensitivity of flies expressing *PxFMO2* to this compound. In contrast to the results for chlorantraniliprole, flies expressing this gene showed no significant resistance to flubendiamide compared to the control line (Fig. 2B). Thus, *PxFMO2* appears to selectively confer resistance to anthranilic diamides.

### 3.5 Evidence of a fitness cost associated with *PxFMO2* overexpression

**Fig. 3:**
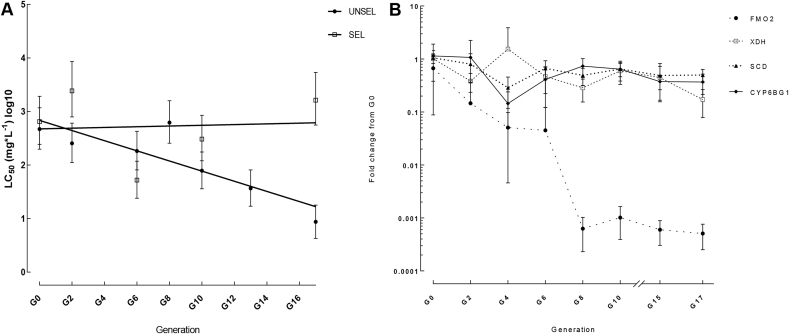
**Chlorantraniliprole susceptibility and expression of candidate resistance genes in selected and unselected lines of the HAW *P. xylostella* strain over the course of 17 generations.** A) Calculated LC_50_ values for chlorantraniliprole for the two lines over the course of the experiment. Error bars indicate 95% CLs. B) Expression of candidate resistance genes in the two lines over the experiment as measured by QPCR. Error bars indicate SD.

During culturing and bioassay of the HAW strain, it became apparent that the level of resistance of this strain may be unstable in the absence of selection suggesting a possible fitness cost associated with resistance. To explore this further the HAW line was split into two cultures. One of these was selected with a maintenance dose of chlorantranilprole (6 ppm) every 2nd or 3rd generation while the other line was left unselected. The sensitivity of the two lines to chlorantraniliprole was assessed using insecticide bioassays approximately every other generation over 17 generations (>1 year). As shown in Fig. 3A, the selected line showed no significant shift in sensitivity at generation zero and the conclusion of the experiment at G17. In contrast, the LD_50_ of the unselected population showed a 52-fold decrease from 469 ppm [95% Cl 198, 1170] at G0 to 9 ppm [95% Cl 4, 18] at G17. To examine if there was any correlation of falling resistance in the unselected line with changes in the expression of candidate resistance genes QPCR was used to test the expression of *PxFMO2*, *PxXDH*, *PxSCD*, and *CYP6BG1* on material preserved at regular intervals during the experiment (Fig. 3B). Only relatively small decreases in the expression of *PxXDH* (−5.9-fold), *PxSCD* (−2.1-fold) and *CYP6BG1* (−3.1-fold) were observed between samples of G0 and G17. In contrast, a 1313-fold reduction in the expression of *PxFMO2* was observed between samples of these two generations. It is worth noting that the decline in expression of *PxFMO2* over the course of the experiment did not show a perfect linear relationship with declining sensitivity to chlorantraniliprole raising the possibility that additional (unknown) mechanisms of resistance may also contribute to the resistance in this strain. Nevertheless, these findings suggest that overexpression of *PxFMO2* in the HAW strain carries a significant fitness cost, and also provides additional evidence that overexpression of *PxFMO2*, but not the other candidate genes, is a causal mechanism of resistance to chlorantraniliprole in this strain.

### 3.6 Alterations in the *PxFMO2* promoter of the HAW strain enhance expression

**Fig. 4:**
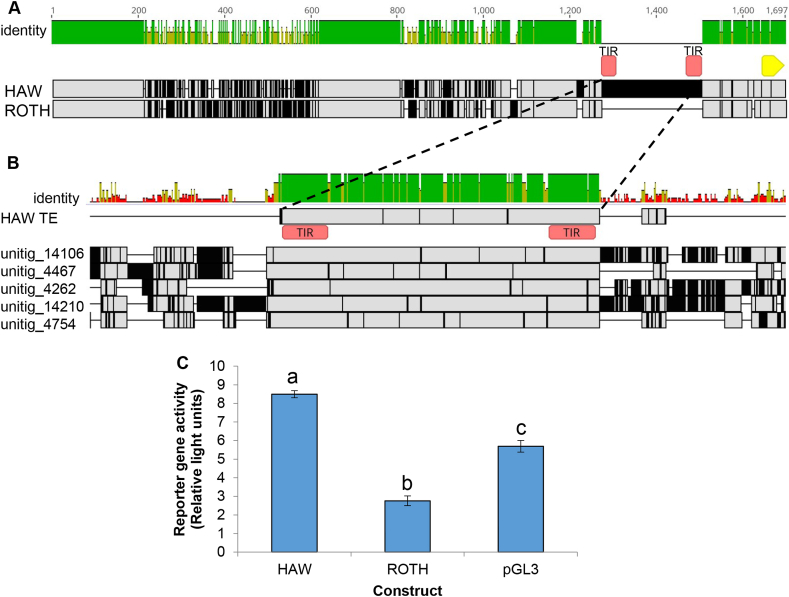
**Alterations in the *PxFMO2* promoter of the HAW strain enhance expression.** A) Alignment of the sequences immediately upstream of *PxFMO2* derived from the HAW and ROTH strains. The terminal inverted repeats (TIR) present at the boundaries of a putative transposable element insertion are indicated in red. The start of the coding sequence of PxFMO2 is shown in yellow. B) Alignment of the PxFMO2 genome against 5 representative scaffolds taken from the *P. xylostella* genome that share the repetitive element. In both A and B grey regions indicate similarity between sequences and black regions indicate sequence differences. Indels are represented by gaps in the sequence. The identity plot above the alignment displays the identity across all sequences for every position. Green indicates that the residue at the position is the same in all sequences. Yellow indicates less than complete identity and red refers to very low identity for the given position. C) Reporter gene activity (normalised to renilla fluorescence) of the ROTH and HAW *PxFMO2* promoter variants. Letters above bars indicate significant differences, P < 0.001; one-way ANOVA with post-hoc Tukey HSD. Error bars indicate 95% CLs. (For interpretation of the references to colour in this figure legend, the reader is referred to the Web version of this article.)

To explore the genetic basis of *PxFMO2* expression in the HAW strain, the ~1500 bp region upstream of the start codon of the gene was characterised from this strain and the ROTH strain by PCR and sequencing. Alignment of the sequences revealed significant divergence between the putative promoters of the two strains with only 63.8% shared sequence identity (Fig. 4A). Both SNPs and indels were observed between the two promoter sequences with the most significant difference resulting from a 233 bp insertion in the HAW promoter sequence just 140 bp upstream of the start codon of *PxFMO2* which was absent in the ROTH promoter (Fig. 4A and B). When this sequence was aligned by BLAST against the *P. xylostella* genome, high scoring hits were returned from >200 scaffolds indicative of a highly repetitive sequence. Furthermore, the boundaries of all copies of this element were found to be defined by 34 bp imperfect terminal inverted repeats (Fig. 4B). Together these findings strongly suggest that this element is a non-autonomous transposon that has inserted into the upstream region of the *PxFMO2* gene in the HAW strain, however, no hits against known transposable element sequences in the NCBI nr or RepBase databases were recovered in BLAST searches, precluding further *in silico* characterisation. To examine the functional significance of the genetic variation observed in the promoter of the HAW strain on expression of *PxFMO2* a ~1.5 kb region of the promoter of this strain and the corresponding region of the ROTH strain were cloned into the reporter gene vector pGL3 and transfected into a lepidopteran (Sf9) cell line. Reporter gene assays showed that the HAW promoter insert drove around 3-fold greater reporter gene expression than the ROTH promoter insert (Fig. 4C). This finding provides evidence that the genetic alterations observed in the promoter of the HAW strain are, at least in part, responsible for the overexpression of this gene in the HAW strain.

## 4 Discussion

The primary mechanism of resistance to diamide insecticides in *P. xylostella* populations described to date has been target-site based, and the role, if any, of alternative mechanisms in resistance to this class of insecticides has remained unclear (Troczka et al., 2017). Our data demonstrate that metabolic mechanisms can confer resistance to the diamide chlorantranilprole in *P. xylostella* and highlight a novel enzyme family that has not been previously implicated in resistance to this insecticide class. Transcriptome profiling of diamide resistant and susceptible strains of *P. xylostella* identified several detoxification enzymes overexpressed in the resistant strains. Of the overexpressed genes encoding detoxification enzymes two were particularly highly expressed in the HAW strain which we show lacks the mutations previous shown to confer target-site resistance to diamide insecticides. The first of these, the P450 CYP6BG1, has been implicated in resistance to chlorantraniliprole previously (Li et al., 2018) (see introduction). However, when we expressed this P450 in *D. melanogaster*, we observed no change in the tolerance of transgenic flies to chlorantraniliprole compared to flies lacking the transgene. This contrasts with work by Li et al. (2018) who used the same expression system to show that flies expressing CYP6BG1 (GAL4 > UAS-CYP6BG1) exhibit 26.3% and 20.4% lower mortality than the UAS-CYP6BG1 and Actin-GAL4 parental lines (Li et al., 2018). There are several potential reasons for this discrepancy. First, in our study adult flies were tested against a range of insecticide concentrations and the data were used to calculate LC_50_ values. In contrast, Li et al. (2018) tested larvae against a single concentration of chlorantraniliprole. Second, in our study flies expressing *CYP6BG1* were compared to flies of the same genetic background but without the transgene (progeny of a cross of the UAS driver line minus the transgene and the GAL4 expressing line) whereas Li et al. (2018) compared their *CYP6BG1* expressing line to flies of a different genetic background (the parental UAS-CYP6BG1 and Actin-GAL4 lines). *CYP6BG1* was first shown to be overexpressed in *P. xylostella* strains with resistance to the pyrethroid permethrin (Bautista et al., 2009) and it is possible that the high expression of this P450 in the strains tested in both our study and that of Li et al. (2018) are a result of prior exposure to this class of insecticides. Additional evidence that *CYP6BG1* does not play a role in chlorantraniliprole resistance in the HAW strain was provided by work exploring the stability of resistance in the absence of selection. While over 17 generations the resistance of the unselected line declined more than 50-fold the level of expression of *CYP6BG1* showed only a 3-fold reduction in expression.

The second gene highly overexpressed in the HAW strain encodes a FMO. Transgenic expression of this enzyme in Drosophila made flies significantly more resistant to chlorantranilprole than flies of the same genetic background but without the transgene providing strong evidence of a causal role in resistance. Furthermore, in the absence of selection the expression of *PxFMO2* declined by > 1300-fold in the HAW strain. This finding both provides additional evidence of the causal role of *PxFMO2* in resistance and reveals a potential fitness cost associated with the overexpression of this gene*. PxFMO2* was also overexpressed in the CHL and FLU strains from Thailand relative to a susceptible strain from the same country, but at much lower levels than observed in the HAW strain. Both the CHL and FLU strains are fixed for the G4946E mutation which has been shown to confer high levels of resistance to diamide insecticides. In this context the overexpression of *PxFMO2* likely plays a minor role in resistance. In future it would be useful to examine the relative frequency and distribution of this mechanism in field populations of *P. xylostella* in Hawaii and worldwide. This is especially important given that the strains used in our study were selected in the laboratory, which does not always accurately mimic selection (and the evolution of resistance) in the field (ffrench-Constant, 2013). Thus, the extent to which *PxFMO2* overexpression is observed in strains collected directly from the field and the implications of this for control using diamide insecticides requires further investigation.

Metabolic resistance to insecticides has been most frequently linked to three well characterised families of enzymes, namely P450s, GSTs, and CEs (Li et al., 2007). In contrast, the role of FMOs in insecticide resistance, and in insects more generally, is poorly understood. FMOs have been previously implicated in resistance to insecticides in just one insect species. Work on beet armyworm, *Spodoptera exigua*, described modest increases (1.5-fold) in FMO activity and synergism with the FMO inhibitor methimazole (synergism ratio = 1.9) in a strain with resistance to metaflumizone a voltage-dependent sodium channel blocker (Tian et al., 2014). In a follow on study *SeFMO1*, *SeFMO2* and *SeFMO3* were found to be overexpressed in the metaflumizone resistant strain compared to a susceptible strain, and functional expression of the three FMOs, followed by indirect assessment of their metabolic capacity, suggested they have activity against metaflumizone and lambda-cyhalothrin (Tian et al., 2018). Beyond these studies the primary focus of research on insect FMOs has been investigation of their role in detoxification of plant secondary metabolites. Research on both arctiid moths and the grasshopper *Zonocerus variegatus* has shown that FMOs detoxify pyrrolizidine alkaloids produced by certain plants as an anti-herbivore defence mechanism (Langel and Ober, 2011; Sehlmeyer et al., 2010; Wang et al., 2012). In contrast to the paucity of information on insect FMOs, their functional role in vertebrates is much more clearly understood where they catalyse the oxygenation of a wide range of xenobiotics, including therapeutic drugs, dietary-derived compounds and even certain pesticides (Phillips and Shephard, 2008). Vertebrate FMOs have been shown to have a preference for nucleophilic substrates that contain nitrogen or sulfur including amines and amides (Ziegler, 2002). The metabolism of chlorantraniliprole, a nitrogen rich carboxamide, by PxFMO2 is thus consistent with the substrate preference of previously characterised FMOs. Interestingly, while the HAW strain showed high resistance to this compound, it showed only low levels of resistance to the diamide flubendiamide. This was correlated with a lack of resistance to flubendiamide in transgenic flies expressing *PxFMO2* to this compound. Thus, PxFMO2 appears to selectively confer resistance to anthranilic diamides but no, or much lower, resistance to phthalic compounds.

Investigation into the molecular basis of *PxFMO2* overexpression revealed a range of genetic alterations in the promoter of this gene in the HAW strain compared to the susceptible ROTH strain, including a putative transposable element insertion in the proximal promoter region. The promoter sequence derived from the HAW strain significantly increased the expression of a reporter gene relative to that of the ROTH strain in a lepidopteran cell line, suggesting the alterations identified explain, at least in part, the enhanced expression of *PxFMO2* in the HAW strain. However, the difference in expression driven by the HAW and ROTH promoters in reporter assays does not reflect the profound difference in the expression of *PxFMO2* seen in the HAW and ROTH *P. xylostella* strains. This suggests that alternative *cis*- or *trans-*acting factors may also play a role in *PxFMO2* overexpression in the HAW strain. Alternatively, the non-native cell line used (from *Spodoptera frugiperda*) may not fully recapitulate the regulatory factors that control the expression of *PxFMO2* in *P. xylostella*. It would be interesting in future studies to examine the expression of deletion constructs of the *PxFMO2* promoter from the HAW strain, including those with and without the repetitive element in order to confirm that this is the bona fide *cis*-acting mutation that results in overexpression. If so this would provide yet another example of the adaptive role of transposable elements in modifying gene expression when inserted near genes. From an applied perspective it would also provide a marker linked to *PxFMO2* overexpression that could be used to develop diagnostics with which to monitor for this resistance mechanism in the field. Finally, previous work on *S. exigua* showed that exposure to several insecticides, including chlorantraniliprole, induces the expression of *SeFMO1-3* (Tian et al., 2018). Given this finding it will be interesting in future to explore the potential of diamide insecticides to induce *PxFMO2* and establish the relative importance of induced versus constitutive mechanisms in the upregulation of this gene in the HAW strain.

In conclusion, we describe a novel mechanism of resistance to the diamide insecticide chlorantraniliprole and provide new evidence that insect FMOs can be recruited to provide protection against synthetic insecticides.

## Appendix A Supplementary data

Table S1 Sequences of primers used in this study. Primer sequences are listed along with the purpose for which they were used Table S1

Tables S2–S4 Transcripts identified as significantly differentially expressed in three different microarray comparisons of diamide resistant and susceptible *P. xylostella* strains. Each comparison is shown in a separate tab - in the first comparison the ROTH strain was compared to the HAW strain, in the second comparison the CM strain was compared to the CHL strain and in the third comparison the CM strain was compared to the FLU strain. The full list of transcripts with functional annotation is provided along with Log2, calculated fold-change values, description based on the closest BLAST hit, and the sequence of each transcript on which probes were designed. Tables S2–S4
