# Supplementary material for: A flavin-dependent monooxgenase confers resistance to chlorantraniliprole in the diamondback moth, Plutella xylostella
Source: Insect Biochem Mol Biol. 2019 Dec;115:103247. doi: 10.1016/j.ibmb.2019.103247 (PMC6880784; doi:10.1016/j.ibmb.2019.103247)
Supplement: Tables S2–S4 — Transcripts identified as significantly differentially expressed in three different microarray comparisons of diamide resistant and susceptible P. xylostella strains. Each comparison is shown in a separate tab - in the first comparison the ROTH strain was compared to the HAW strain, in the second comparison the CM strain was compared to the CHL strain and in the third comparison the CM strain was compared to the FLU strain. The full list of transcripts with functional annotation is provided along with Log2, calculated fold-change values, description based on the closest BLAST hit, and the sequence of each transcript on which probes were designed. [file mmc2.docx]

| Primer name | Sequence | Purpose |
| --- | --- | --- |
| PxGADPH F1 | CCCTTCATCGGTCTGGACTA | QPCR amplification of the GADPH reference gene |
| PxGADPH R1 | CAGCGATCTTGTTTCCGTT | QPCR amplification of the GADPH reference gene |
| PxActin F2 | TCGGTATGGGACAGAAGGAC | QPCR amplification of the actin reference gene |
| PxActin R2 | AGGTGTGGTGCCAGATCTTC | QPCR amplification of the actin reference gene |
| PxTubulin F1 | ATCCCGAACAACGTCAAAAC | QPCR of the tubulin reference gene |
| PxTubulin R1 | AGCTCTTGGATTGCTGTCGT | QPCR of the tubulin reference gene |
| 39731_Solute carrier F1 | GGTTCCTCTCTTGACTCTGTGC | QPCR of sugar transporter contig_39731 |
| 39731_Solute carrier F2 | CGAACGCTAGCAGCCAAT | QPCR of sugar transporter contig_39731 |
| 33985_CE F1 | CCGTAGAGATGCGGCTAAAG | QPCR of caboxylesterase contig_33985 |
| 33985_CE R1 | AGGTGAACGACGGATAGACG | QPCR of caboxylesterase contig_33985 |
| 42842_FMO2 F2 | GGAAGACATGATGGCGAAGT | QPCR of PxFMO2 |
| 42842_FMO2 R2 | TCTTGTACGTGCCTGACTCG | QPCR of PxFMO2 |
| AB372008.1_CYP6BG1 F2 | ATG TTG TTA TTG GTA GCT CTG GT | QPCR of CYP6BG1 |
| AB372008.1_CYP6BG1 R2 | TGT GGC GGA AAC ATA GAG AGT | QPCR of CYP6BG1 |
| 1528_XDH F2 | AGGGCTGCATACAAAAATGG | QPCR of xanthine dehydrogenase contig_1528 |
| 1528_XDH R2 | GAAAGCAGCATTGACCCAGT | QPCR of xanthine dehydrogenase contig_1528 |
| 42163_SCD_F1 | GAGGAACTAGGACGCCCTTC | QPCR of short chain dehydrogenase contig_42163 |
| 42163_SCD_R2 | GATCGCTGGCCAGATAAACA | QPCR of short chain dehydrogenase contig_42163 |
| 2422_UGT_F2 | AGCGAGAACATCCTTTTGCG | QPCR of UDP-glucuronosyltransferase contig_2422 |
| 2422_UGT_R2 | CGGTGCTGATGAAATGGGA | QPCR of UDP-glucuronosyltransferase contig_2422 |
| FMO_CDS_F5 | ATGTTATTTAAACACTCCATAATTTTGTG | Sequence verification of PxFMO2 coding sequence |
| FMO_CDS_R3 | CTGGCCGTGTATCTTCTGC | Sequence verification of PxFMO2 coding sequence |
| FMO_CDS_R1 | ATG ATT TGT AGG ACT GCA GCT A | Sequence verification of PxFMO2 coding sequence |
| XDH_CDS_F | ATG TCC ATG TAC ACT TTA CTG C | Sequence verification of xanthine dehydrogenase coding sequence |
| XDH_CDS_R | CT AAG GCA CAA TGT TCC ATG G | Sequence verification of xanthine dehydrogenase coding sequence |
| SCD_CDS_F5 | ATGGACTTCAACAACAAAGTCGT | Sequence verification of short chain dehydrogenase coding sequence |
| SCD_CDS_R5 | CATCAATGATTCACCGTTATCGA | Sequence verification of short chain dehydrogenase coding sequence |
| SCD_CDS_R6 | CAGCATCCTTGATCGGTTTGA | Sequence verification of short chain dehydrogenase coding sequence |
| CYP6BG1_CDS_F1 | GCTATCGCGTGCGTCTGTA | Sequence verification of CYP6BG1 coding sequence |
| CYP6BG1_CDS_R3 | CAATACTGAGTGGAAGAACGC | Sequence verification of CYP6BG1 coding sequence |
| CYP6BG1_CDS_F4 | GGGATCAACACCGGAGTCA | Sequence verification of CYP6BG1 coding sequence |
| CYP6BG1_CDS_R4 | GAACCGCATTCCTATACATATTCTGGG | Sequence verification of CYP6BG1 coding sequence |
| CYP6BG1_CDS_F9 | TCCGAGTCCACATCCCCAT | Sequence verification of CYP6BG1 coding sequence |
| CYP6BG1_CDS_R9 | TATAGAGTAACCCTCCTCGTC | Sequence verification of CYP6BG1 coding sequence |
| Px_FMOProm_F1 | GTCAAAATCTGCACCTCG | PCR amplification of the PxFMO2 promoter region |
| Px_FMOProm_R1 | ACACTAGTCAAGAAGCACA | PCR amplification of the PxFMO2 promoter region |
| Px_FMOProm_SacI_F2 | aattgagctcGAGTGCATGAAGTTTGA | Cloning of PxFMO2 promoter region into PGL3 |
| Px_R_FMOPromNcoI_R2 | aattccatggATAACATTTTGAACTCTT | Cloning of PxFMO2 promoter region into PGL3 |
| Px_HS_FMOPromNcoI_R2 | aattccatggATAACATTTTGCACTCTT | Cloning of PxFMO2 promoter region into PGL3 |
| D099 pUAST F | TCACTGGAACTAGGCTAGCA-3' | Sequence validation of transgenic flies |
| D102 pUAST F | 5'-GGATCCAAGCTTGCATGCCTG-3' | sequence validation of transgenic flies |
| D100 pUAST R | 5'-AAAGGCATTCCACCACTGCT-3' | sequence validation of transgenic flies |
| D101 pUAST R | 5'-CCACCACTGCTCCCATTCAT-3' | sequence validation of transgenic flies |
